# Endothelin-1 is associated with fibrosis in proliferative diabetic retinopathy membranes

**DOI:** 10.1371/journal.pone.0191285

**Published:** 2018-01-19

**Authors:** William Chang, Michelle Lajko, Amani A. Fawzi

**Affiliations:** Department of Ophthalmology, Northwestern University, Feinberg School of Medicine, Chicago, IL, United States of America; Cedars-Sinai Medical Center, UNITED STATES

## Abstract

**Purpose:**

To characterize the relationship between endothelin-1 and fibrosis in epiretinal membranes in proliferative diabetic retinopathy and explore the role of endothelial-mesenchymal transition in these membranes.

**Methods:**

Membranes were obtained from eyes undergoing pars plana vitrectomy for complicated proliferative diabetic retinopathy or idiopathic epiretinal membrane. Through standard immunohistochemical techniques, we labeled membranes to explore the distribution of endothelin-1 and endothelin receptor B, comparing proliferative diabetic retinopathy and idiopathic epiretinal membranes. In addition, membranes were also labeled with markers for fibroblasts, endothelial, and glial cells and studied with confocal laser scanning microscopy. The intensity of endothelin-1 labeling was quantified using standard image analysis software.

**Results:**

Fourteen membranes were included in the analysis, nine from eyes with proliferative diabetic retinopathy and five idiopathic membranes. Flatmount diabetic membranes showed co-localization of endothelin-1 with S100A4 and CD31. Immunohistochemistry and quantitative analysis of cross-sectional membranes showed significantly higher endothelin-1 labeling in proliferative diabetic retinopathy membranes compared to idiopathic membranes (p<0.05). Diabetic membranes showed more elements staining positive for S100A4 compared to idiopathic membranes.

**Conclusion:**

Epiretinal membrane formation in proliferative diabetic retinopathy involves higher tissue levels of endothelin-1 and fibroblastic activity. Furthermore, endothelin-1, endothelial and fibroblastic staining appear to be correlated, suggestive of endothelial-to-mesenchymal transition in proliferative diabetic retinopathy.

## Introduction

Diabetic retinopathy (DR) is a major cause of blindness worldwide and is prevalent in almost 80% of diabetics after 10 years of diabetes [1]. Pericytes, involved in maintaining the blood-retinal barrier, are affected early in DR, compromising the endothelial cells and initiating a cascade of microvascular changes including microaneurysms and capillary occlusion in early stages of non-proliferative DR [2]. Proliferative diabetic retinopathy (PDR) is a late stage manifestation of DR, where progressive retinal ischemia leads to elaboration of angiogenic factors, including VEGF, with ultimate growth of new blood vessels along the interface between the retina and the vitreous humor. In PDR, angiogenesis and subsequent fibrosis on the retinal surface often result in the formation of fibrovascular, tractional epiretinal membranes, which lead to vision threatening complications such as tractional retinal detachment and ultimately vision loss [2–5].

While the clinical manifestations of advanced DR are well established, the underlying molecular and biochemical mechanisms remain poorly understood. Recent studies have shown that the epiretinal membranes and vitreous of patients with PDR contain different protein marker patterns than non-diabetic tissues [6–10]. One such molecule is endothelin (ET), which has been shown to be higher in the vitreous of PDR patients compared to non-diabetic vitreous [11, 12]. Endothelin is a potent vasoconstrictor, which exists in three isoforms: ET-1, ET-2, and ET-3, of which ET-1 is the most abundant form recognized by endothelin receptors A (ETA) and B (ETB). In the retina, ET-1 and its receptor ETA have been shown to mediate decreased retinal blood flow during hyperglycemia and in DR [13]. Furthermore, hyperglycemia has been shown to directly increase ET-1 secretion from endothelial cells [14]. In the initial stages of DR, ET-1 appears to mediate pericyte death via ETA receptors [5].

The ability of ET-1 to promote fibrosis has been extensively studied in other pathological conditions, such as pulmonary and cardiac fibrosis [15, 16]. It has been suggested that ET-1 is involved in fibroblast maturation and the deposition of fibrous tissue in lung [17]. A recent study showed that ET-1 and ETB are expressed in cells of human epiretinal membranes in proliferative vitreoretinopathy, another form of epiretinal fibrosis [18]. Similarly, membranes of PDR patients were shown to exhibit diffuse ET-1 expression [3]. Furthermore, DR has been associated with increased ETB expression in diabetic rat retinas [19]. However, the relative expression and role of ET-1 and ETB in PDR membranes compared to idiopathic ERMs has not been studied. While most studies have focused on the expression of ET-1 in PDR fibrovascular membranes, few have explored the relationship between ET-1 expression and fibroblast proliferation within these epiretinal membranes.

Recent studies have identified endothelial-to-mesenchymal transition (EndoMT) as a potential mechanistic pathway that is involved in fibrosis [10, 20–23]. In this process, endothelial cells lose their apical-basal polarity along with endothelial cell markers such as CD31. These endothelial cells transform into mesenchymal cells and take on fibroblastic markers such as S100A4. EndoMT has been demonstrated in several organ systems including cardiac and kidney fibrosis [24, 25]. Furthermore, ET-1 has been suggested to be a mediator in EndoMT in disease processes such as systemic sclerosis [26].

In this study, we investigate the hypothesis that ET-1 plays a role in promoting fibrosis through EndoMT in PDR. We explore the association between ET-1 and fibroblast proliferation in both diabetic and idiopathic epiretinal membranes. We studied EndoMT using immunohistochemical techniques comparing the localization of the fibroblastic marker S100A4 with the endothelial marker CD31 as well as with ET-1. We also compared the relative immunofluorescence of ETB with glial fibrillary acidic protein (GFAP) in both diabetic and idiopathic epiretinal membranes in an effort to further characterize their potential roles in membrane formation.

## Methods

The study protocol and procedures adhered to the ethics tenets of the Declaration of Helsinki and were approved by the Institutional Review Board at Northwestern University. Epiretinal membranes were removed from eyes undergoing routine surgery and pars plana vitrectomy (PPV). Verbal informed patient consent was obtained prior to surgery. Surgeries were routine 23-gauge PPV without the use of adjuvant dyes. Idiopathic membranes were peeled from the retinal surface with microsurgical forceps, while PDR membranes required a combination of segmentation with the vitreous cutter followed by removal of the segmented free membranes with microsurgical forceps. Excised membranes were designated as either from PDR or as idiopathic based on the patient history and underlying surgical diagnosis. All membranes were fixed in a 4% paraformaldehyde for 24 hours and then transferred to a 30% sucrose solution for 5 hours.

### Frozen cross-sections: Tissue preparation and immunohistochemistry

Membranes were embedded in optimal cutting temperature compound (OCT). Tissue sections (7 μm) were thawed, washed with tris-buffered saline (TBS), and blocked with 10% normal donkey serum for one hour at room temperature. Samples were incubated with primary antibody (Table 1) diluted in blocking solution at 4°C for 18 hours. Following several TBS washes, sections were incubated with donkey anti-goat rhodamine red (705-295-147, Jackson ImmunoResearch, West Grove, PA) or donkey anti-rabbit Alexa Fluor 594 (ab96921, Abcam, Cambridge, UK) secondary antibody for one hour at room temperature. Isolectin IB4 (I21413, Thermo Fisher Scientific) was used as a vascular marker in lieu of a primary antibody. Sections were then washed with TBS and stained with DAPI (4’, 6’-diamino-2-phenylindole) to stain nuclei. In several immunostains, SYTOX Green nucleic acid stain (S7020, Thermo Fisher Scientific, Waltham, MA) replaced DAPI for nuclei staining because of microscope limitations. Slides were mounted with ProLong Gold Antifade reagent (P36930, Thermo Fisher Scientific). Each immunostain was performed with a negative control using the secondary antibody without primary antibody. These negative controls showed faint and nonspecific staining.

**Table 1:** Primary antibody profile.

Primary Antibody	Dilution	Source	Manufacturer
ET-1	1:400	Rabbit	Peninsula (T-4050)
ETB	1:200	Rabbit	Antibody Research Corporation*
S100A4	1:400	Rabbit	Abcam (ab27957)
GFAP	1:200	Rabbit	Abcam (ab7260)
CD31	1:100	Rabbit	Abcam (ab28364)

Abbreviations: ET-1, endothelin-1; ETB, endothelin receptor B; GFAP, glial fibrillary acidic protein.

*ETB antibody was custom made by Antibody Research Corporation, St. Charles, MO.

Immunofluorescence images were obtained using a Nikon A1R+ Confocal Laser Microscope System (Minato, Japan). Single plane immunofluorescent images were obtained at total magnification of 600x.

### Flatmount immunohistochemistry and co-localization studies

Three additional diabetic epiretinal membranes were prepared as flatmounts. These membranes were fixed in 4% paraformaldehyde (Electron Microscopy Sciences, Hatfield, PA) diluted in phosphate buffered saline (PBS) for 3–4 hours. The membranes were transferred to 30% sucrose until staining. After several washes, membranes were permeabilized with PBS/0.5% Triton X-100 for 18 hours at 4°C. The membranes were blocked with 10% donkey serum, 0.1% Triton X-100, 1% bovine serum albumin for 5 hours. Rabbit anti-S100A4 (Abcam, Cambridge, UK, ab27957) diluted 1:300 and goat anti-ET-1 (Santa Cruz, Dallas, TX, sc-21625) diluted 1:75 or rat anti-CD31 (BD Biosciences, San Jose, CA, 553370) diluted 1:50 were applied for 18 hours at 4°C in blocking solution. After several washes, the membranes were incubated in donkey anti-rabbit Rhodamine Red (Jackson ImmunoResearch, West Grove, PA, 711-295-152, 1:300 dilution), donkey anti-goat Alexa Fluor 488 (Jackson ImmunoResearch, 705-545-147, 1:200 dilution), or goat anti-rat Alexa Fluor 488 (Thermo Fisher Scientific, Waltham, MA, A-11006, 1:200 dilution) diluted in PBS/0.1% Triton X-100 for 18 hours at 4°C. After several washes, the membranes were counterstained with DAPI (Thermo Fisher Scientific) for 2 minutes, washed, mounted with ProLong Gold Antifade reagent (Thermo Fisher Scientific), and sealed.

### Quantification of ET-1 immunofluorescence

Immunofluorescence images were obtained using the Zeiss LSM 510 laser scanning confocal microscope (Oberkochen, Germany). Single plane immunofluorescence and differential interference contrast (DIC) images were obtained for each membrane at a total magnification of 100x. Microscope settings were kept constant for all imaging. Image analysis was performed using Fiji software (NIH, Bethesda, MD) [27]. Image stitching was performed on several images in order to incorporate an entire membrane section in one image. Tissue outlines were detected via the “Find Edges” plugin function in Fiji software using the DIC image for a given tissue. The image was thresholded and converted into a binary image. The “analyze particles” function was used to apply a mask that represented the area of the membrane on brightfield, which was subsequently used for the corresponding immunofluorescent image. The pixel intensity of the selected area of the immunofluorescent image was measured. The intensities of idiopathic and PDR membranes were measured and recorded. An unpaired two tailed Student’s t-test was performed using SPSS Statistics. (Version 23.0; IBM Corporation, New York, USA).

## Results

### Patient demographics

A total of 14 epiretinal membranes were obtained from nine patients with PDR and five patients with idiopathic membranes. Two PDR membranes were prepared as flatmounts. One PDR membrane was excluded from the analysis because the patient was on hydroxychloroquine treatment, a medication that has been shown to result in increased autofluorescence of retinal tissue [28]. Demographic and clinical data are shown in Table 2 and Table 3.

**Table 2:** Demographic characteristics of patients with proliferative diabetic retinopathy epiretinal membranes.

ERM Number	Age/Sex	Diabetes Type	Duration of Diabetes (yrs)	HbA1c	Past Medical History
P1	44/M	1	39	7.6	ESRD
P2	53/M	2	9	8.3	Esophageal cancer, smoking
P3	51/F	1	>30	8.1	None
P4	39/F	2	15	7	None
P5	30/F	1	17	13.9	Pregnant at time of surgery for repair traction retinal detachment and ERM removal
P6	29/M	1	18	8	Asthma
P7	53/F	2		6.4	Hypertension, coronary artery disease, asthma
P8	33/F	1	>25	7.2	None
P9	45/M	2	20	8.2	Hypertension, asthma

Abbreviations: ERM, epiretinal membrane; ESRD, end-stage renal disease; HbA1C, hemoglobin A1C.

**Table 3:** Demographic characteristics of patients with idiopathic epiretinal membranes.

ERM Number	Age/Sex	Visual Symptom Duration	Past Medical History
E1	57/F	3 months	None
E2	71/M	3 months	None
E3	44/M	2 years	Kjer's optic neuropathy
E4	72/F	5 years	Hypertension, hyperlipidemia
E5	65/M	1 year	None

Abbreviations: ERM, epiretinal membrane.

### ET-1, S100A4, and CD31 immunoreactivity in flatmount membranes

**Fig 1:**
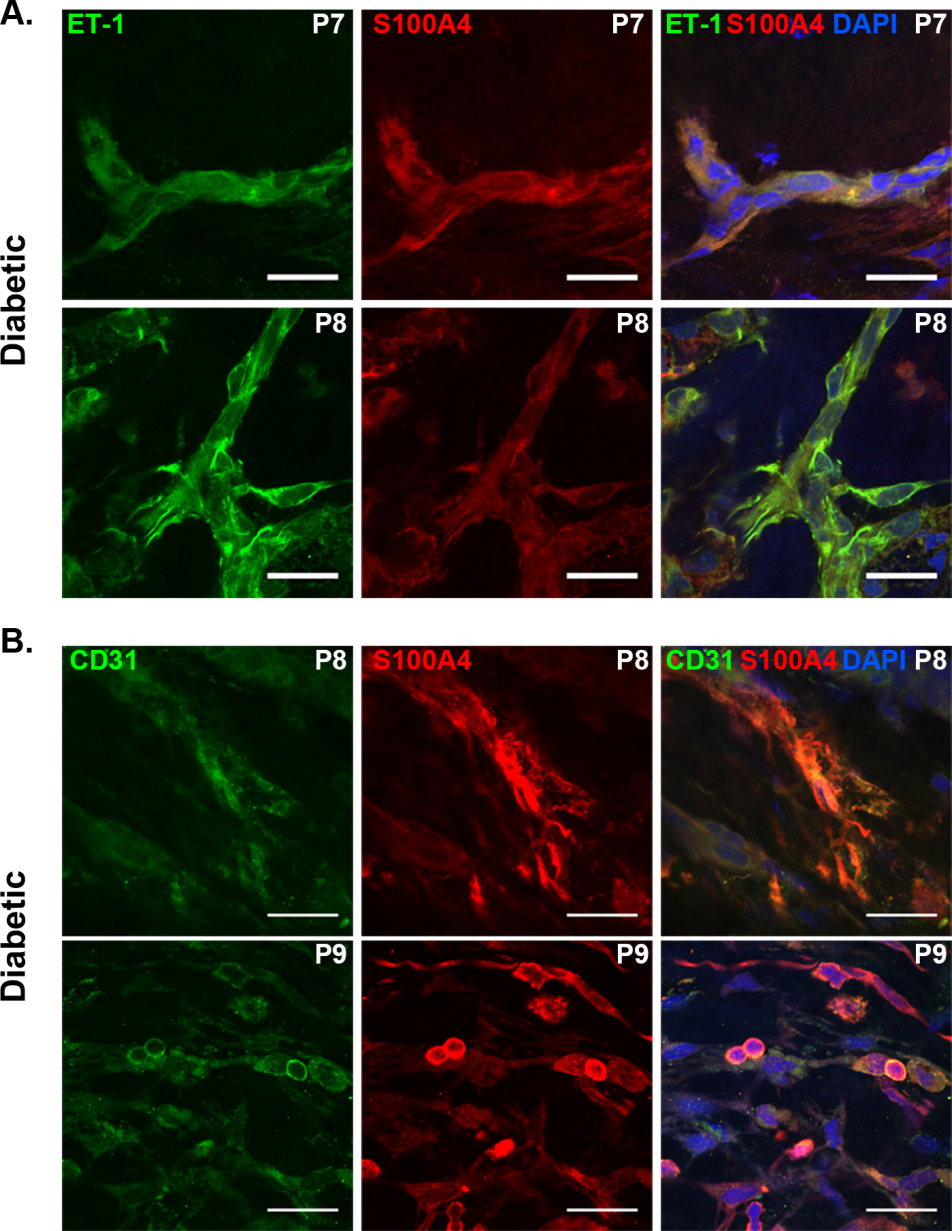
Co-localization of endothelin-1 (ET-1) with S100A4 and CD31. Panel A shows merged confocal images of ET-1 (green) and S1004A (red) immunofluorescence in flatmount proliferative diabetic retinopathy epiretinal membranes. Panel B shows merged confocal images of co-localization of ET-1 (green) and CD31 (red) immunofluorescence in flatmount proliferative diabetic retinopathy epiretinal membranes. Nuclei are labeled with DAPI (blue). Patient numbers are indicated in the upper-right corners. Scale bars: 25 μm.

Two flatmount PDR membranes were analyzed and shown to have co-localization of ET-1 and S100A4 immunofluorescence (Fig 1, P7, P8). In addition, co-localization of S100A4 and CD31 was also observed in PDR membranes (Fig 1, P8, P9).

### S100A4 immunoreactivity in cross sections

**Fig 2:**
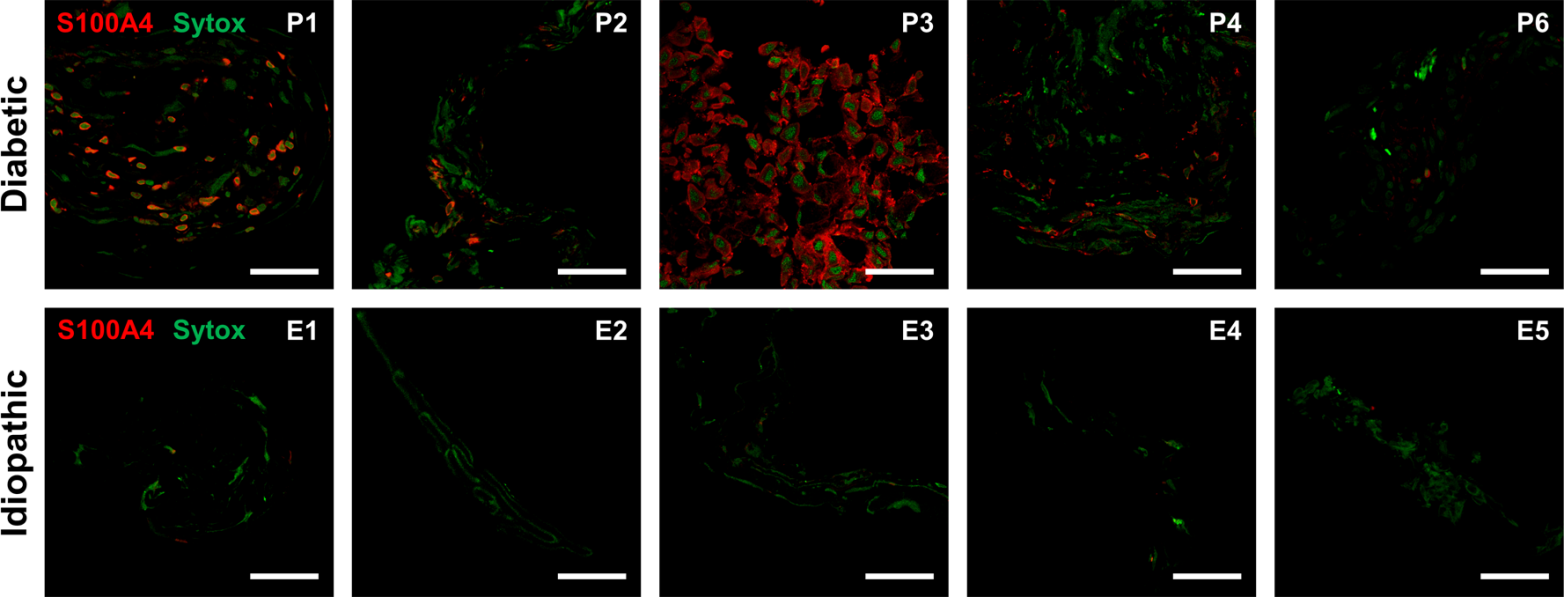
Fibroblast (S100A4) immunofluorescence in cross-sectional proliferative diabetic retinopathy and idiopathic epiretinal membranes. Patient numbers are indicated in the upper-right corners. Confocal images show diabetic epiretinal membranes (top) have more S100A4-positive cells (red) than idiopathic epiretinal membranes (bottom). Nuclei are labeled with SYTOX Green nuclear stain (green). Scale bars: 50 μm.

Identification of fibroblasts was performed by immunostaining with S100A4. Idiopathic epiretinal membranes showed little immunoreactivity with S100A4 and generally had less cellular components within the membrane (Fig 2). However, PDR membranes had variable S100A4 immunoreactivity. While some PDR epiretinal membranes exhibited strong and widespread cellular S100A4 labeling (Fig 2, P1, P2), others showed more limited immunoreactivity (Fig 2, P3, P6).

### ET-1 immunoreactivity in cross sections

**Fig 3:**
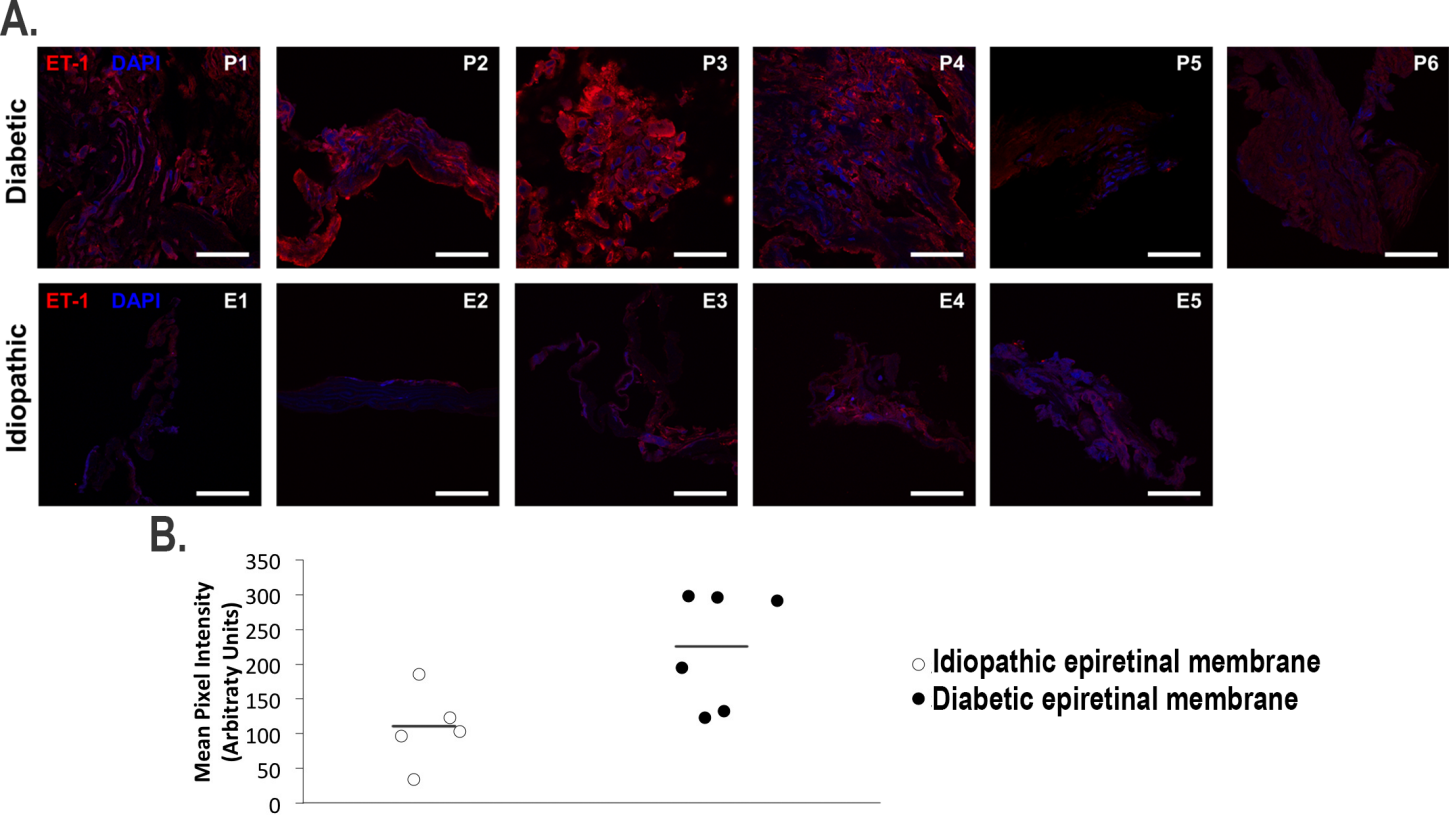
Endothelin-1 (ET-1) immunofluorescence in cross-sectional proliferative diabetic retinopathy and idiopathic epiretinal membranes. Panel A shows confocal images of individual diabetic epiretinal membranes (top) show moderate ET-1 (red) staining throughout the tissue. Confocal images of idiopathic epiretinal membranes (bottom) showed weaker ET-1 immunofluorescence compared to diabetic epiretinal membranes. Nuclei are labeled with DAPI (blue). Patient numbers are indicated in the upper-right corners. Scale bars: 50 μm. Panel B shows quantification of ET-1 immunofluorescence in cross-sectional idiopathic and proliferative diabetic retinopathy epiretinal membranes. Pixel intensity was measured in arbitrary units (AU). Black bars represent the mean pixel intensity for the given type of epiretinal membrane.

**Fig 4:**
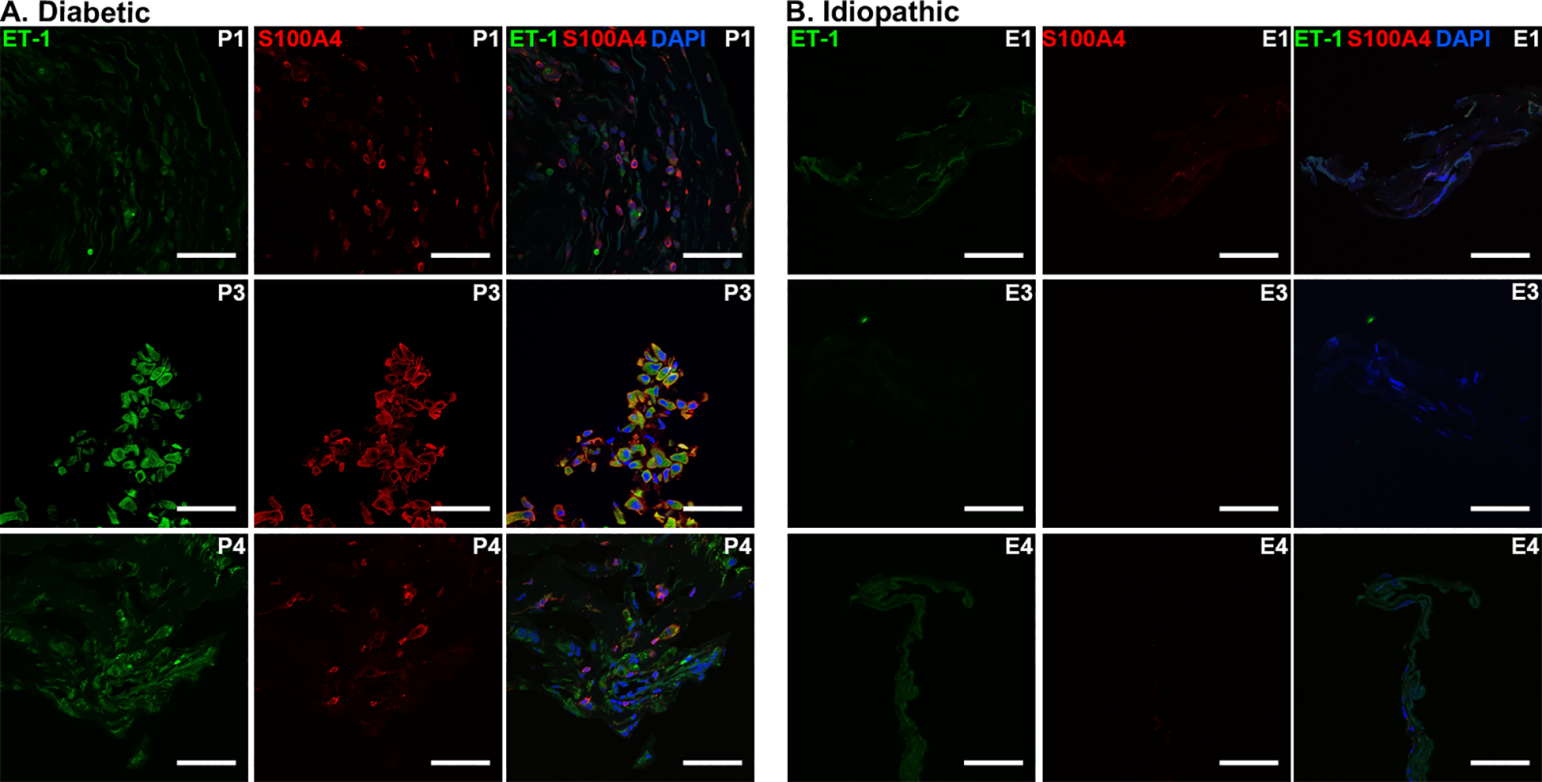
Co-localization of Endothelin-1 (ET-1) and S100A4 in representative cross-sectional proliferative diabetic retinopathy and idiopathic epiretinal membranes. Merged confocal images of diabetic ERMs (A) show co-localization of ET-1 (green) and S100A4 (red). Nuclei are labeled with DAPI (blue). Patient numbers are indicated in the upper-right corners. Scale bars: 50 μm.

Confocal microscopy analysis of PDR epiretinal membranes showed moderate ET-1 immunostaining throughout the tissue (Fig 3, Panel A). Idiopathic membranes generally showed less ET-1 immunostaining compared to diabetic epiretinal membranes. In addition, merged images with DAPI show that ET-1 was localized to the cytoplasm of these cells. Quantitative analysis of ET-1 immunofluorescence (Fig 3, Panel B) showed that PDR membranes had significantly higher mean pixel intensity (222.4 ± 30.9 arbitrary units) than idiopathic epiretinal membranes (108.5 ± 21.8 arbitrary units; unpaired t-test, p<0.05). When double immunostaining was performed with S100A4, ET-1 was observed to co-localize with S100A4 in diabetic epiretinal membranes (Fig 4).

### ETB immunoreactivity in cross sections

**Fig 5:**
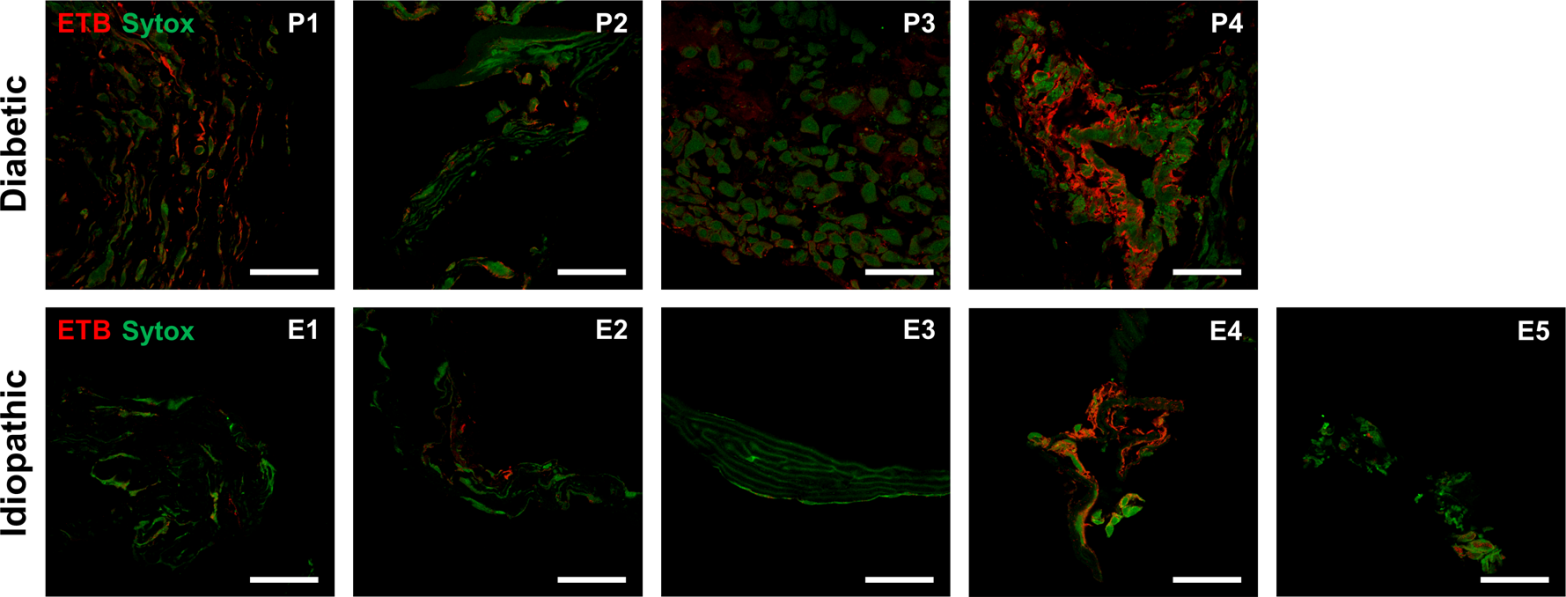
Endothelin receptor B (ETB) immunofluorescence in cross-sectional proliferative diabetic retinopathy and idiopathic epiretinal membranes. Confocal images of diabetic ERMs (top) show variable ETB (red) staining among tissue samples. Confocal images of idiopathic ERMs (bottom) also showed variable ETB immunofluorescence. Nuclei are labeled with SYTOX Green nuclear stain (green). Patient numbers are indicated in the upper-right corners. Scale bars: 50 μm.

ETB was detected in both PDR and idiopathic ERMs. Analysis via confocal microscopy showed variable levels of ETB immunofluorescence throughout PDR and idiopathic membranes (Fig 5). Of the diabetic membranes, P1 and P4 had the most prominent ETB staining, while E4 showed the most prominent ETB staining among the idiopathic membranes.

### GFAP immunoreactivity in cross sections

**Fig 6:**
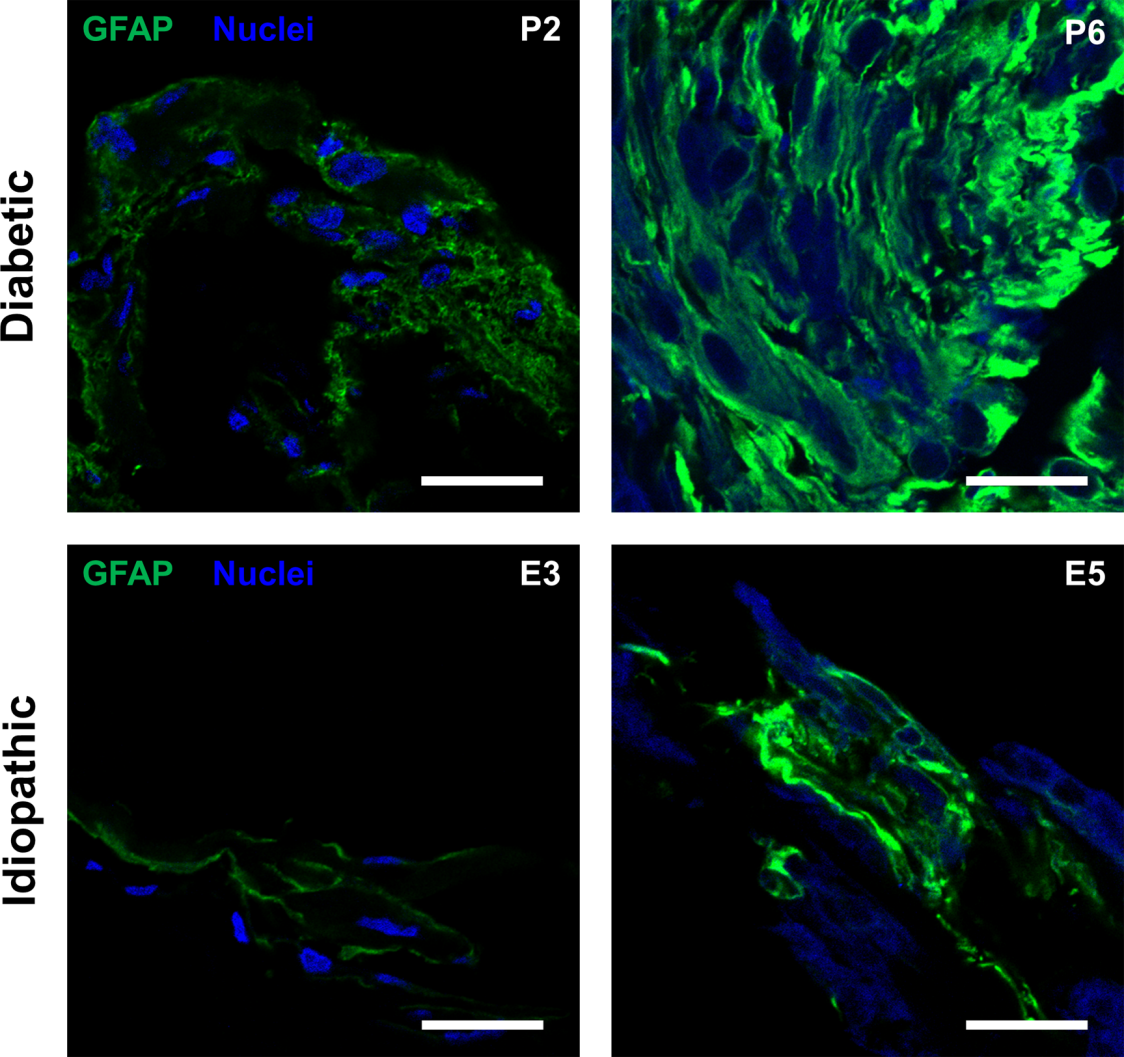
Glial fibrillary acid protein (GFAP) immunofluorescence representative cross-sectional proliferative diabetic retinopathy and idiopathic epiretinal membranes. Confocal images of both diabetic (top) and idiopathic ERMs (bottom) show GFAP (green) immunostaining within the tissue stroma. Nuclei are labeled with DAPI (blue). Patient numbers are indicated in the upper-right corners. Scale bars: 25 μm.

Glial cellular components were detected through immunostaining with GFAP. Both idiopathic and diabetic ERMs had variable GFAP immunofluorescence. Diabetic patients P2 and P6, along with idiopathic patients E3 and E5, showed particularly strong GFAP immunofluorescence (Fig 6). In patients E3 and E5, GFAP is seen in only portions of the membranes while other areas are devoid of GFAP staining. However, in patients P2 and P6, GFAP immunofluorescence is more diffuse.

### IB4 and CD31 immunoreactivity in cross sections

CD31 immunoreactivity was detected in a flatmount diabetic ERM as shown in Fig 1. In addition, CD31 was observed to co-localize with S100A4 in these flatmount ERMs. However, cross-sectional immunohistochemistry of PDR and idiopathic ERMs failed to show specific endothelial cell staining with IB4 or CD31 antibodies.

## Discussion

Fibrovascular membrane formation in PDR involves the proliferation of fibroblasts alongside regressing abnormal vasculature, with eventual contraction of these membranes leading to retinal detachment [29]. In general, diabetic membranes showed more diffuse staining for fibroblasts, as shown in Fig 2. Idiopathic epiretinal membranes showed minimal S100A4 staining, suggesting that active fibrosis and fibroblastic transformation may not be a prominent feature of membrane formation in these patients. In general, fibrosis is a well-recognized hallmark of advanced PDR; therefore, the intense fibroblast presence in diabetic membranes is not surprising. This is consistent with recent evidence showing increased fibrocyte activity in PDR epiretinal membranes [30]. It is interesting to note that only PDR flatmount tissue showed specific staining for CD31, a marker of endothelial cells. This is in contrast to previous studies that demonstrated a relatively high number of endothelial cells in PDR membranes. There are several potential explanations for these diverse findings, including the fact that blood vessels are only sparsely distributed in these membranes and may be missed in cross-section preparations. Another potential explanation relates to the process of EndoMT, characterized by the loss of endothelial vascular markers along with the acquisition of fibroblastic markers, which could explain the variation in the expression of vascular markers such as CD31. Membranes with relatively sparse CD31 may represent a more advanced stage in the EndoMT process. When single sections were used for immunostaining, it is possible that areas with CD31 staining were missed. Flatmounted membranes were more likely to demonstrate both the overall distribution of cellular markers as well as individual blood vessels. We used the cross-sectional approach, in addition to flatmounting, to maximize the number of sections and the range of antibodies and molecular stains we can apply to each individual membrane.

In immunostained flatmount diabetic ERMs, we identified vessels that were immunolabeled with both ET-1 and S100A4 (Fig 1, P7, P8). Furthermore, we identified cellular elements that co-expressed both CD31 and S100A4 (Fig 1, P8, P9). These findings highlight the possibility that vasculature within these membranes are undergoing a transition towards fibrosis, via EndoMT. Fibrosis does not appear to be separate process from endothelial cell dysfunction but is considered a maladaptive process in various ischemic and vascular diseases, such as pulmonary hypertension and vein graft remodeling [31, 32]. In patients with PDR, we believe that these endothelial cells are intimately involved and may transdifferentiate to give rise to the proliferation of fibroblasts [20–23].

The co-localization of CD31, ET-1 and S100A4 seen in Figs 1 and 4 suggests that endothelial cell dysfunction is related to fibroblast proliferation in PDR, and supports the potential for endoMT in this process. Studies have shown that fibrosis can be associated with EndoMT as well as transdifferentiation of several cell types, including pericytes and epithelial cells [33]. It is possible that the diabetic ERMs in this study represent the latter stages of EndoMT in PDR, which is characterized by the diminishing presence of the vascular network as endothelial cells transition towards a fibroblastic phenotype [23, 34]. This process of transition can also explain the variable expression of S100A4, CD31 and ET-1 seen in diabetic ERMs. In one study, ET-1 treatment of endothelial cells resulted in increased transformation to a fibroblast state [26].

In this study, we demonstrate diffuse staining for ET-1 in diabetic and idiopathic membranes, where immunofluorescence quantification showed significantly higher intensity of ET-1 in PDR membranes (Fig 3). Our results suggest that, while ET-1 may be important in mediating fibrosis in various retinal pathologies, this role is particularly heightened in PDR. While the pathophysiology of fibrous tissue formation in PDR is likely complex and currently understudied, previous studies have shown that ET-1 is upregulated in cardiac fibrosis in diabetics as well as in ischemic myocardial tissue [24, 35, 36]. However, it remains unclear if ET-1 found in these diabetic membranes is produced from the cells within the membranes. One study determined that ET-1 is expressed in cells derived from PDR membranes [37]. Other studies have established that both serum and vitreous ET-1 levels are higher in diabetic patients than in non-diabetics [3]. Thus, the source of ET-1 found in ERMs may be a combination of retinal origin and also the systemic circulation.

As shown in Fig 5, ETB was detected in both idiopathic and diabetic membranes. Interestingly, some diabetic membranes exhibited intense staining while others showed minimal staining. Similar variation was present in the idiopathic membranes, where ETB could be seen in particular zones of the tissue rather than throughout the entire tissue section. Compared to the cytoplasmic localization of ET-1, ETB appears to localize predominantly to the periphery of the cell, likely the cell membrane. This localization of ETB is expected given that the receptor is a transmembrane G protein-coupled receptor [38]. In diabetic membranes, the level of ET-1 immunofluorescence did not qualitatively correlate with the level of ETB immunostaining. Several diabetic epiretinal membranes with high mean pixel intensities of ET-1 immunofluorescence had fairly mild ETB immunofluorescence (Fig 5, P2, P3). This discrepancy is difficult to interpret given the complexity of interactions between ET-1 and ETB [39, 40].

The presence of GFAP immunostaining in both PDR and idiopathic epiretinal membranes (Fig 6) suggests that glial cells, such as retinal Müller cells and astrocytes, are a prominent component of these membranes. Indeed, it has been shown that glial cells, particularly retinal Müller cells have been found in idiopathic and diabetic epiretinal membranes [41–43]. Müller cells are believed to upregulate GFAP expression in conditions involving retinal traction [44]. Because this mechanical stress likely occurs in both idiopathic and diabetic epiretinal membrane formation, it is not surprising that both types of epiretinal membranes exhibit increased GFAP. The relatively higher immunostaining of ET-1 and S100A4 in PDR epiretinal membranes may be an indication that PDR epiretinal membranes arise via different mechanisms than idiopathic epiretinal membranes. The precursor events to PDR epiretinal membranes often involve complications such as angiogenesis and hemorrhage secondary to retinal vasculature damage. These membranes are usually intricately associated with regressing and active neovascular fronds growing on the posterior vitreous face. The resulting membrane formation could arise secondary to the release of fibrotic markers from the damaged endothelium or as we hypothesize, through EndoMT, the transition of endothelial cells to active fibroblasts. However, additional markers such type I collagen and α-SMA are needed to provide confirmatory evidence.

The patient characteristics were highly heterogeneous in this study, which poses several challenges in interpreting the composition of the epiretinal membranes, including the age difference between the two groups, with diabetic patients being overall younger in age. While this may represent a potential bias in patient selection, the diabetic patients often presented with epiretinal membranes after years of having diabetes and usually in the setting of advanced proliferative disease with exuberant angiogenesis. The epiretinal membranes formed in PDR are simply the result of a disease process that typically presents earlier in life. Idiopathic epiretinal membranes were typically seen in older patients, suggesting that the process driving their formation is not substantial until later in life. We believe the mechanisms for DR and idiopathic membranes are potentially different based on the differences observed in immunostaining. However, we cannot rule out subtle mechanistic similarities given that these membranes and our studies are essentially snapshots of a dynamic and chronic disease process. While it is possible that idiopathic membranes are derived from patients who present early in their disease course (thus the smaller membranes with fewer cells), the complications of diabetic retinopathy likely account for the increase in fibrotic components seen in diabetic ERMs. Another important distinction in the pathogenesis of these two entities relates to the tight relationship between fibrosis and angiogenesis in diabetic membranes, a temporal and spatial relationship that suggests an intricate process that entangles the two pathologic mechanisms, with endoMT as one potential uniting pathomechanism. Concomitant medical conditions, ocular treatments, and duration of disease could potentially affect the properties of their membranes. Diabetic patients tended to have more frequent health maintenance visits than patients with idiopathic membranes. In general, diabetic epiretinal membranes were larger in size compared to idiopathic membranes. While this may reflect a difference in the underlying molecular mechanisms of membrane formation, symptom characteristics may have also contributed. Patients with idiopathic membranes typically presented with mild blurring of the vision and metamorphopsia as their visual complaint. These patients could attribute their main symptom with the formation of an epiretinal membrane. In contrast, diabetic patients presented with several ocular morbidities related to DR, many of which preceded the formation of the membrane. Most of these eyes underwent surgery to repair acute on chronic traction retinal detachment. These diabetic patients may have presented only after becoming severely symptomatic or with a large, longstanding epiretinal membrane and decreased vision due to progression of their traction detachment into the macula.

Lack of membrane orientation is a limitation to this study. Membranes peeled from the retinal surface often lost their orientation during processing making it close to impossible to determine the vitreal or retinal side. Thus, during cryosectioning, it was not possible to determine the exact geometry in which the epiretinal membrane was sectioned. In addition, several idiopathic and diabetic epiretinal membranes yielded a limited number of sections because of the small size of the tissue. This posed a logistical hurdle as several epiretinal membranes could not be immunostained with all antibody targets due to the limited number of sections.

Sample size and patient heterogeneity are other limitations to this study. Patients were recruited on a rolling basis. Patients who progress to traction detachment often have developed other significant clinical complications of diabetic detachment and these patients had prior ocular treatments for their PDR, including laser and injection therapies, which may complicate the molecular content of the eye and epiretinal membranes. We excluded from this study diabetic eyes that had undergone extensive treatment with anti-angiogenic medications. In addition, several membranes could not be successfully retrieved intact and could not be included in this study. However, the differences between the two study groups remained statistically significant, even with this small sample size.

### Strengths

Our study focused on membranes collected using the same surgical protocol (single surgeon), which did not use adjuvant dyes for staining. Overall, we used standardized approaches for immunostaining with emphasis on appropriate controls, with staining of idiopathic and diabetic membranes at the same time using the same reagents. This allowed for more consistent measurements of pixel intensities across all samples, especially for quantification of ET-1. We used an automated image analysis approach to define tissue boundaries based on DIC imagery for ET-1 quantification. This protocol increased the objectivity and confidence of the quantification analysis [45].

In conclusion, we have shown that ET-1 and fibroblast immunostaining are more evident in PDR than in idiopathic membranes. These findings support the hypothesis that ET-1 is upregulated in advanced PDR and may be an important player in the pathophysiology of fibroblastic transitions. In addition, the co-localization of ET-1 and S100A4 staining suggests that ET-1 may be linked to fibroblastic proliferation in PDR. We also demonstrate co-localization of CD31 and S100A4 in these diabetic membranes, suggestive of the transformative process associated with EndoMT. Because of the differences in cellular composition, we believe epiretinal membranes in PDR arise from different mechanisms than idiopathic epiretinal membranes. Since this was a pilot study, we believe that quantitative measurements, including protein and RNA analysis are important aspects for future studies, which are currently underway. Further work is required to better delineate the exact pathophysiologic mechanism by which ET-1 promotes the transition towards fibroblast phenotype and to explore whether ET-1 could present a viable therapeutic target to address and perhaps prevent fibroblastic transformation and tractional retinal detachments in PDR.

## References

[1] Kertes PJ, Johnson TM. Evidence-based eye care. Philadelphia, PA: Lippincott Williams & Wilkins; 2007 xiv, 338 p. p.

[2] Ciulla TA, Amador AG, Zinman B. Diabetic retinopathy and diabetic macular edema: pathophysiology, screening, and novel therapies. Diabetes Care. 2003;26(9):2653–64. 12941734 10.2337/diacare.26.9.2653

[3] Roldan-Pallares M, Rollin R, Martinez-Montero JC, Fernandez-Cruz A, Bravo-Llata C, Fernandez-Durango R. Immunoreactive endothelin-1 in the vitreous humor and epiretinal membranes of patients with proliferative diabetic retinopathy. Retina. 2007;27(2):222–35. doi: 10.1097/01.iae.0000231376.76601.40 17290206 10.1097/01.iae.0000231376.76601.40

[4] Oberstein SY, Byun J, Herrera D, Chapin EA, Fisher SK, Lewis GP. Cell proliferation in human epiretinal membranes: characterization of cell types and correlation with disease condition and duration. Mol Vis. 2011;17:1794–805. 21750605 PMC3133557

[5] Chou JC, Rollins SD, Ye M, Batlle D, Fawzi AA. Endothelin receptor-A antagonist attenuates retinal vascular and neuroretinal pathology in diabetic mice. Investigative ophthalmology & visual science. 2014;55(4):2516–25. 24644048 10.1167/iovs.13-13676 PMC4585571

[6] Demircan N, Safran BG, Soylu M, Ozcan AA, Sizmaz S. Determination of vitreous interleukin-1 (IL-1) and tumour necrosis factor (TNF) levels in proliferative diabetic retinopathy. Eye (Lond). 2006;20(12):1366–9. 16284605 10.1038/sj.eye.6702138

[7] Gao BB, Chen X, Timothy N, Aiello LP, Feener EP. Characterization of the vitreous proteome in diabetes without diabetic retinopathy and diabetes with proliferative diabetic retinopathy. J Proteome Res. 2008;7(6):2516–25. doi: 10.1021/pr800112g 18433156 10.1021/pr800112g

[8] Lim JI, Spee C, Hinton DR. A comparison of hypoxia-inducible factor-alpha in surgically excised neovascular membranes of patients with diabetes compared with idiopathic epiretinal membranes in nondiabetic patients. Retina. 2010;30(9):1472–8. doi: 10.1097/IAE.0b013e3181d6df09 20811317 10.1097/IAE.0b013e3181d6df09 PMC3690285

[9] Yoshida S, Ishikawa K, Asato R, Arima M, Sassa Y, Yoshida A, et al Increased expression of periostin in vitreous and fibrovascular membranes obtained from patients with proliferative diabetic retinopathy. Invest Ophthalmol Vis Sci. 2011;52(8):5670–8. doi: 10.1167/iovs.10-6625 21508107 10.1167/iovs.10-6625

[10] Vereb Z, Lumi X, Andjelic S, Globocnik-Petrovic M, Urbancic M, Hawlina M, et al Functional and molecular characterization of ex vivo cultured epiretinal membrane cells from human proliferative diabetic retinopathy. Biomed Res Int. 2013;2013:492376 doi: 10.1155/2013/492376 24195074 10.1155/2013/492376 PMC3806336

[11] Adamiec-Mroczek J, Oficjalska-Mlynczak J, Misiuk-Hojlo M. Roles of endothelin-1 and selected proinflammatory cytokines in the pathogenesis of proliferative diabetic retinopathy: Analysis of vitreous samples. Cytokine. 2010;49(3):269–74. doi: 10.1016/j.cyto.2009.11.004 20015663 10.1016/j.cyto.2009.11.004

[12] Zhou J, Wang S, Xia X. Role of intravitreal inflammatory cytokines and angiogenic factors in proliferative diabetic retinopathy. Curr Eye Res. 2012;37(5):416–20. doi: 10.3109/02713683.2012.661114 22409294 10.3109/02713683.2012.661114

[13] Wang Z, Yadav AS, Leskova W, Harris NR. Attenuation of streptozotocin-induced microvascular changes in the mouse retina with the endothelin receptor A antagonist atrasentan. Experimental eye research. 2010;91(5):670–5. doi: 10.1016/j.exer.2010.08.008 20727883 10.1016/j.exer.2010.08.008 PMC2962698

[14] Park JY, Takahara N, Gabriele A, Chou E, Naruse K, Suzuma K, et al Induction of endothelin-1 expression by glucose: an effect of protein kinase C activation. Diabetes. 2000;49(7):1239–48. 10909984 10.2337/diabetes.49.7.1239

[15] Swigris JJ, Brown KK. The role of endothelin-1 in the pathogenesis of idiopathic pulmonary fibrosis. BioDrugs. 2010;24(1):49–54. doi: 10.2165/11319550-000000000-00000 20055532 10.2165/11319550-000000000-00000 PMC2855311

[16] Leask A. Potential therapeutic targets for cardiac fibrosis: TGFbeta, angiotensin, endothelin, CCN2, and PDGF, partners in fibroblast activation. Circulation research. 2010;106(11):1675–80. doi: 10.1161/CIRCRESAHA.110.217737 20538689 10.1161/CIRCRESAHA.110.217737

[17] Ross B, D'Orleans-Juste P, Giaid A. Potential role of endothelin-1 in pulmonary fibrosis: from the bench to the clinic. Am J Respir Cell Mol Biol. 2010;42(1):16–20. doi: 10.1165/rcmb.2009-0175TR 19717811 10.1165/rcmb.2009-0175TR

[18] Iribarne M, Ogawa L, Torbidoni V, Dodds CM, Dodds RA, Suburo AM. Blockade of endothelinergic receptors prevents development of proliferative vitreoretinopathy in mice. Am J Pathol. 2008;172(4):1030–42. doi: 10.2353/ajpath.2008.070605 18310504 10.2353/ajpath.2008.070605 PMC2276418

[19] Chakrabarti S, Gan XT, Merry A, Karmazyn M, Sima AA. Augmented retinal endothelin-1, endothelin-3, endothelinA and endothelinB gene expression in chronic diabetes. Current eye research. 1998;17(3):301–7. 9543639 10.1076/ceyr.17.3.301.5216

[20] Abu El-Asrar AM, De Hertogh G, van den Eynde K, Alam K, Van Raemdonck K, Opdenakker G, et al Myofibroblasts in proliferative diabetic retinopathy can originate from infiltrating fibrocytes and through endothelial-to-mesenchymal transition (EndoMT). Exp Eye Res. 2015;132:179–89. doi: 10.1016/j.exer.2015.01.023 25637870 10.1016/j.exer.2015.01.023

[21] Cao Y, Feng B, Chen S, Chu Y, Chakrabarti S. Mechanisms of endothelial to mesenchymal transition in the retina in diabetes. Invest Ophthalmol Vis Sci. 2014;55(11):7321–31. doi: 10.1167/iovs.14-15167 25335984 10.1167/iovs.14-15167

[22] Quaggin SE, Kapus A. Scar wars: mapping the fate of epithelial-mesenchymal-myofibroblast transition. Kidney international. 2011;80(1):41–50. doi: 10.1038/ki.2011.77 21430641 10.1038/ki.2011.77

[23] Piera-Velazquez S, Mendoza FA, Jimenez SA. Endothelial to Mesenchymal Transition (EndoMT) in the Pathogenesis of Human Fibrotic Diseases. J Clin Med. 2016;5(4). 10.3390/jcm5040045 PMC4850468 27077889

[24] Widyantoro B, Emoto N, Nakayama K, Anggrahini DW, Adiarto S, Iwasa N, et al Endothelial cell-derived endothelin-1 promotes cardiac fibrosis in diabetic hearts through stimulation of endothelial-to-mesenchymal transition. Circulation. 2010;121(22):2407–18. doi: 10.1161/CIRCULATIONAHA.110.938217 20497976 10.1161/CIRCULATIONAHA.110.938217

[25] Zeisberg EM, Potenta SE, Sugimoto H, Zeisberg M, Kalluri R. Fibroblasts in kidney fibrosis emerge via endothelial-to-mesenchymal transition. J Am Soc Nephrol. 2008;19(12):2282–7. doi: 10.1681/ASN.2008050513 18987304 10.1681/ASN.2008050513 PMC2588112

[26] Cipriani P, Di Benedetto P, Ruscitti P, Capece D, Zazzeroni F, Liakouli V, et al The Endothelial-mesenchymal Transition in Systemic Sclerosis Is Induced by Endothelin-1 and Transforming Growth Factor-beta and May Be Blocked by Macitentan, a Dual Endothelin-1 Receptor Antagonist. J Rheumatol. 2015;42(10):1808–16. doi: 10.3899/jrheum.150088 26276964 10.3899/jrheum.150088

[27] Schindelin J, Arganda-Carreras I, Frise E, Kaynig V, Longair M, Pietzsch T, et al Fiji: an open-source platform for biological-image analysis. Nat Methods. 2012;9(7):676–82. doi: 10.1038/nmeth.2019 22743772 10.1038/nmeth.2019 PMC3855844

[28] Mititelu M, Wong BJ, Brenner M, Bryar PJ, Jampol LM, Fawzi AA. Progression of hydroxychloroquine toxic effects after drug therapy cessation: new evidence from multimodal imaging. JAMA Ophthalmol. 2013;131(9):1187–97. doi: 10.1001/jamaophthalmol.2013.4244 23887202 10.1001/jamaophthalmol.2013.4244

[29] Hiscott P, Hagan S, Heathcote L, Sheridan CM, Groenewald CP, Grierson I, et al Pathobiology of epiretinal and subretinal membranes: possible roles for the matricellular proteins thrombospondin 1 and osteonectin (SPARC). Eye (Lond). 2002;16(4):393–403. 12101446 10.1038/sj.eye.6700196

[30] Tamaki K, Usui-Ouchi A, Murakami A, Ebihara N. Fibrocytes and Fibrovascular Membrane Formation in Proliferative Diabetic Retinopathy. Invest Ophthalmol Vis Sci. 2016;57(11):4999–5005. doi: 10.1167/iovs.16-19798 27661853 10.1167/iovs.16-19798

[31] Ranchoux B, Antigny F, Rucker-Martin C, Hautefort A, Pechoux C, Bogaard HJ, et al Endothelial-to-mesenchymal transition in pulmonary hypertension. Circulation. 2015;131(11):1006–18. doi: 10.1161/CIRCULATIONAHA.114.008750 25593290 10.1161/CIRCULATIONAHA.114.008750

[32] Cooley BC, Nevado J, Mellad J, Yang D, St Hilaire C, Negro A, et al TGF-beta signaling mediates endothelial-to-mesenchymal transition (EndMT) during vein graft remodeling. Science translational medicine. 2014;6(227):227ra34 doi: 10.1126/scitranslmed.3006927 24622514 10.1126/scitranslmed.3006927 PMC4181409

[33] Rockey DC, Bell PD, Hill JA. Fibrosis—a common pathway to organ injury and failure. N Engl J Med. 2015;372(12):1138–49. doi: 10.1056/NEJMra1300575 25785971 10.1056/NEJMra1300575

[34] Sanchez-Duffhues G, Orlova V, Ten Dijke P. In Brief: Endothelial-to-mesenchymal transition. The Journal of pathology. 2016;238(3):378–80. doi: 10.1002/path.4653 26446982 10.1002/path.4653

[35] Tonnessen T, Giaid A, Saleh D, Naess PA, Yanagisawa M, Christensen G. Increased in vivo expression and production of endothelin-1 by porcine cardiomyocytes subjected to ischemia. Circ Res. 1995;76(5):767–72. 7728993 10.1161/01.res.76.5.767

[36] Wang X, Guo Z, Ding Z, Khaidakov M, Lin J, Xu Z, et al Endothelin-1 upregulation mediates aging-related cardiac fibrosis. J Mol Cell Cardiol. 2015;80:101–9. doi: 10.1016/j.yjmcc.2015.01.001 25584774 10.1016/j.yjmcc.2015.01.001

[37] Kim LA, Wong LL, Amarnani DS, Bigger-Allen AA, Hu Y, Marko CK, et al Characterization of cells from patient-derived fibrovascular membranes in proliferative diabetic retinopathy. Molecular vision. 2015;21:673–87. 26120272 PMC4462955

[38] Mazzuca MQ, Khalil RA. Vascular endothelin receptor type B: structure, function and dysregulation in vascular disease. Biochem Pharmacol. 2012;84(2):147–62. doi: 10.1016/j.bcp.2012.03.020 22484314 10.1016/j.bcp.2012.03.020 PMC3358417

[39] Kelland NF, Kuc RE, McLean DL, Azfer A, Bagnall AJ, Gray GA, et al Endothelial cell-specific ETB receptor knockout: autoradiographic and histological characterisation and crucial role in the clearance of endothelin-1. Can J Physiol Pharmacol. 2010;88(6):644–51. doi: 10.1139/Y10-041 20628430 10.1139/Y10-041

[40] Schneider MP, Boesen EI, Pollock DM. Contrasting actions of endothelin ET(A) and ET(B) receptors in cardiovascular disease. Annu Rev Pharmacol Toxicol. 2007;47:731–59. doi: 10.1146/annurev.pharmtox.47.120505.105134 17002597 10.1146/annurev.pharmtox.47.120505.105134 PMC2825895

[41] Zhao F, Gandorfer A, Haritoglou C, Scheler R, Schaumberger MM, Kampik A, et al Epiretinal cell proliferation in macular pucker and vitreomacular traction syndrome: analysis of flat-mounted internal limiting membrane specimens. Retina. 2013;33(1):77–88. doi: 10.1097/IAE.0b013e3182602087 22914684 10.1097/IAE.0b013e3182602087

[42] Mizutani M, Gerhardinger C, Lorenzi M. Muller cell changes in human diabetic retinopathy. Diabetes. 1998;47(3):445–9. 9519752 10.2337/diabetes.47.3.445

[43] Bu SC, Kuijer R, van der Worp RJ, Huiskamp EA, Renardel de Lavalette VW, Li XR, et al Glial cells and collagens in epiretinal membranes associated with idiopathic macular holes. Retina. 2014;34(5):897–906. doi: 10.1097/IAE.0000000000000013 24077090 10.1097/IAE.0000000000000013

[44] Guidry C. The role of Muller cells in fibrocontractive retinal disorders. Progress in retinal and eye research. 2005;24(1):75–86. doi: 10.1016/j.preteyeres.2004.07.001 15555527 10.1016/j.preteyeres.2004.07.001

[45] Jensen EC. Quantitative analysis of histological staining and fluorescence using ImageJ. Anat Rec (Hoboken). 2013;296(3):378–81. 23382140 10.1002/ar.22641

